# Evaluating User Experiences and Preferred Features of a Web-Based 24-Hour Dietary Assessment Tool: Usability Study

**DOI:** 10.2196/63823

**Published:** 2024-10-18

**Authors:** Berit Follong, Caitlin Haliburton, Sally Mackay, Maria Maiquez, Jacqueline Grey, Cliona Ni Mhurchu

**Affiliations:** 1 National Institute for Health Innovation University of Auckland Auckland New Zealand; 2 School of Population Health Faculty of Medical and Health Sciences University of Auckland Auckland New Zealand; 3 The Centre for Translational Health Research: Informing Policy and Practice (TRANSFORM) University of Auckland Auckland New Zealand

**Keywords:** public health, nutrition, dietary assessment methods, digital tools, user experience, qualitative data, survey

## Abstract

**Background:**

Intake24, a web-based 24-hour dietary recall tool developed in the United Kingdom, was adapted for use in New Zealand (Intake24-NZ) through the addition of a New Zealand food list, portion size images, and food composition database. Owing to the customizations made, a thorough evaluation of the tool’s usability was required. Detailed qualitative usability studies are well suited to investigate any challenges encountered while completing a web-based 24-hour recall and provide meaningful data to inform enhancements to the tool.

**Objective:**

This study aims to evaluate the usability of Intake24-NZ and identify improvements to enhance both the user experience and the quality of dietary intake data collected.

**Methods:**

We used a mixed methods approach comprising two components: (1) completion of a single 24-hour dietary recall using Intake24-NZ with both screen observation recordings and collation of verbal participant feedback on their experience and (2) a survey.

**Results:**

A total of 37 participants aged ≥11 years self-completed the dietary recall and usability survey (men and boys: 14/37, 38% and women and girls: 23/37, 62%; Māori: 10/37, 27% and non-Māori: 27/37, 73%). Although most (31/37, 84%) reported that Intake24-NZ was easy to use and navigate, data from the recorded observations and usability survey revealed challenges related to the correct use of search terms, search results obtained (eg, type and order of foods displayed), portion size estimation, and associated food prompts (eg, did you add milk to your tea?).

**Conclusions:**

This comprehensive usability study identified challenges experienced by users in completing a dietary recall in Intake24-NZ. The results informed a series of improvements to enhance user experience and the quality of dietary data collected with Intake24-NZ, including adding new foods to the food list, optimizing the search function and ordering of search results, creating new portion size images, and providing clearer instructions to the users.

## Introduction

### Background

Large-scale or national nutrition surveys are essential to monitor population food and nutrient intakes, assess nutritional status, evaluate nutrition programs and policies, and provide evidence for new interventions and strategies [1]. A range of dietary assessment methods are commonly used in such surveys, with the 24-hour dietary recall tool being used most frequently [2,3] and increasing interest and demand driving a transition to automated systems in recent years [4]. Technological advancements have enabled reduced participant burden and costs while providing the ability to capture data from large population samples, standardize the recall process, use digital portion size estimation aids, and automatically match food intakes to an appropriate food composition database [5].

Intake24, a web-based 24-hour dietary recall tool developed in the United Kingdom, was selected for use in a future New Zealand (NZ) Nutrition Survey based on a comprehensive review and evaluation process [6]. The tool was customized to suit the unique needs of the NZ population by adding a new local food list (eg, traditional cultural foods and local food names), updating portion size options and images (eg, including NZ-specific brands of packaged products), and modifying prompts appropriately (eg, reminders of foods commonly consumed together such as butter on bread). A detailed description of the iterative process used to develop the NZ food list has been published elsewhere [6].

Adapting dietary assessment methods, including 24-hour recall tools, for use in a new setting (eg, a different country or population) requires thorough evaluation and validation specific to that context [7], as even slight changes to the tool may impact its performance [8]. Different study designs can be used to assess the ability of recall tools to accurately estimate dietary intakes, of which validation and comparison studies are used most often [1,2]. Although the comparison between dietary data obtained by Intake24 and other (objective) measures provides insight into the size of any measurement error, it does not allow the identification of the type and source of error that is critical to improve the accuracy of the tool [9]. Usability studies can be used to explore how 24-hour dietary recall tools are used and understood by respondents and to investigate challenges encountered while completing the recall, thereby providing meaningful data to identify and improve issues related to the functioning of the recall tool and reporting process [9].

To date, the potential to improve the accuracy of 24-hour dietary recall tools through usability studies has not been used to its full extent, as most such studies test usability using retrospective questionnaires or simple rating scales to quantify the feasibility and acceptability of the tools [1,9]. These evaluation methods require respondents to recall details of difficulties they experienced when completing a lengthy and multistep recall process [10,11] and commonly use closed-ended questions that limit the collection of in-depth feedback [9]. Furthermore, the ability of respondents to accurately identify errors and their related causes is arguable [9].

### Objective

As such, it is essential when aiming to assess usability to deploy detailed qualitative approaches that capture comprehensive and real-time data to inform further improvement of the 24-hour recall tool. We aimed to investigate the usability of the NZ version of Intake24 (Intake24-NZ) using a novel mixed methods approach that involved observations, think-aloud techniques, and a usability survey.

## Methods

### Study Design and Participant Recruitment

This cross-sectional, mixed methods study was conducted between April and June 2023 and comprised two parts: (1) completion of a single 24-hour dietary recall and (2) a usability survey on the user experience of completing the dietary recall using Intake24-NZ. Individuals aged ≥11 years were eligible to participate and were recruited using a combination of targeted convenience and snowball sampling. Potential participants were invited by emailing recruitment materials and study information to the researchers’ networks, including colleagues, wider stakeholders, and people who participated in previous research and expressed interest in being involved in future nutrition-related studies. As this strategy mainly targeted adults, children were recruited through snowball sampling. Adults with children aged 11 to 15 years were asked to invite their children. The recruitment email also included a link to a screening survey that was used to determine individuals’ eligibility and to balance recruitment across age and ethnic groups (Māori [Indigenous people of NZ] vs non-Māori people). Participants were grouped as follows: (1) children aged 11 to 15 years, (2) adults aged 16 to 64 years and completing their own recall, (3) parent proxies aged 16 to 64 years and completing the recall on behalf of their child aged 2 to 10 years, and (4) older adults aged ≥65 years. From the list of eligible individuals, the research team invited those who fitted the age and ethnic group distribution on a first-come, first-served basis while listing the remaining individuals on a waiting list.

### Ethical Considerations

The study was approved by the University of Auckland Human Participants Ethics Committee, NZ (approved on March 15, 2023; UAHPEC25426). Consent forms were sent to the selected individuals, who were required to sign and return the forms before participation in the study. Parental consent and child assent were sought for children aged 11 to 15 years. Assent was not obtained from children aged 2 to 10 years for whom the parents or caregivers completed the dietary recall on their behalf. Although the intake data collected concerned the child, this study aimed to determine the usability of the dietary recall tool, and therefore, only the parent’s or caregiver’s ability to complete the parent proxy recall was of interest. All participants received a NZ $20 (US $12) voucher as a token of appreciation for participating in the study. To protect participant information collected, data were deidentified and stored in a secure web-based folder only accessible to the research team.

### 24-Hour Dietary Recall Tool Development

**Figure 1:**
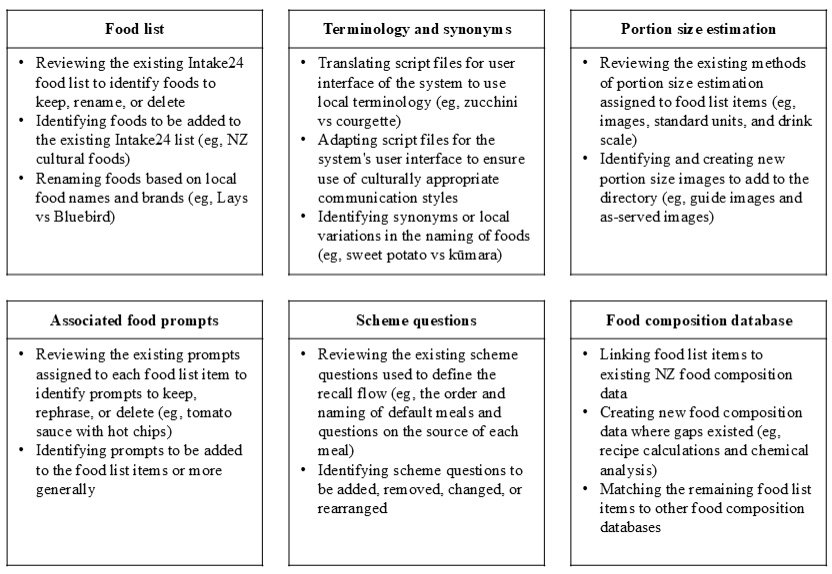
Modifications required to adapt Intake24, a 24-hour dietary recall tool, for use in New Zealand (NZ).

Intake24 is an open-source dietary assessment system originally developed at Newcastle University (United Kingdom) and funded by the Food Standards Agency, Scotland [12-14]. It is now maintained and developed through a collaboration between Newcastle University, Cambridge University (United Kingdom), and Monash University (Australia). The automated web-based tool helps people record their dietary intake for the previous 24 hours by following the steps of a multiple-pass 24-hour recall method. Participants search for foods from a predefined food list and select the amount consumed using a range of portion size estimation aids, such as images, drinking scales, standard measurement units (eg, measuring cups or spoons), categorical size estimates (eg, small, medium, or large), and food units (eg, 1 egg) [12]. Two main types of portion size images are used: as-served and guide images. As-served images display food served on a plate or in a bowl using 7 images showing progressive increases in the portion size (eg, a range of portions of pasta or soup). The amount of food depicted in these 7 images has been validated using adult data from the United Kingdom National Diet and Nutrition Survey, which used weighed food diaries (portions span from the 5th to the 95th percentile of the weighted food) [13]. Guide images display food available in a variety of predetermined amounts (eg, different sizes of tomatoes or bags of chips). The number of portion size images presented to participants differs by food, ranging from no image (ie, alternative portion size estimation aids are used, such as standard measurement units) to 5 options. Participants are prompted about food and drinks commonly consumed together (eg, milk in tea) and commonly forgotten items (eg, water and snacks). A sandwich and salad builder can be used to detail individual ingredients consumed in a sandwich or salad. Other features include an instruction video explaining how to complete a recall, a navigation panel (meal menu) that lists the meals and foods entered by the participants, contextual help buttons, and a *missing foods* function to record food items that cannot be found in the tool’s food list [13,15]. The final steps of the recall require the participant to review their reported food and drink items and to submit the recall. Intake data are then automatically linked to a food composition database to obtain information on the individual’s energy and nutrient intake [6]. Adaptation of Intake24 (version 3, 2022-2023 [16]) for use in NZ required modifying several aspects of the original tool, including the food list, terminology, portion size estimation aids, prompts, food synonyms, and food composition database [6]. An overview of these modifications, including examples, is provided in Figure 1. Multimedia Appendix 1 includes images of the steps in the multiple-pass recall method and other Intake24-NZ functionalities. A report describing the process of adapting the food list and food composition database for NZ has been recently published [6]. A written agreement between our institution and the Medical Research Council Epidemiology Unit, University of Cambridge, was signed, outlining the terms of our collaboration and the scope of the use and modifications to the tool.

### Data Collection

#### Dietary Recall Setup and Instructions

Following consent, participants were contacted to complete a short survey about their sociodemographic characteristics, provide further instructions, and schedule a date for completion of the dietary recall and usability survey. Sociodemographic information collected included age, gender, ethnicity, highest qualification completed, and whether they had a (professional) background in nutrition. Dates for the recall completion were selected by the research team to ensure that these were completed on both weekdays and weekend days. Participants received a calendar invitation, including a Zoom (Zoom Video Communications) link once the date was confirmed. As the purpose of this study was not to assess the accuracy of the dietary intake data, participants were allowed to record their food and drink intake (take notes or photos) on the day before completing the 24-hour dietary recall to help recall the type and amount consumed. Participants were sent email reminders about recording their food intake and to prompt them to complete their recall. Furthermore, participants were emailed a personalized web link for the tool Intake24-NZ and a separate link to a survey about the usability of the tool. Using the personalized web link, participants were directed to the Intake24-NZ platform and asked to record the type and amount of food and drinks consumed during the previous day (midnight to midnight). All participants recorded their own intake, except for adults who completed a parent proxy recall for their child aged 2 to 10 years. Participants received no training on how to use Intake24-NZ, except for a brief 2-minute instructional video that is integrated into the tool. Although participants were requested to self-complete the 24-hour dietary recall, they were allowed to seek help from others or to contact the research team via email, phone, or Zoom for support with Intake24-NZ and technical issues.

#### Observations From Dietary Recall Screen and Voice Recordings

Zoom was used to record participants’ screens and voices while completing the 24-hour dietary recall. They were asked to share their screen and think aloud. By thinking aloud [17], participants were able to verbalize their thoughts when they were confused or surprised, ran into issues, or had any other comments about the tool [13]. Both screen and voice recordings were used by the research team to identify usability issues and gain a better understanding of the user experience and functioning of Intake24-NZ. No recordings of the participants themselves were taken as they were requested to turn off their camera. As the aim was to explore the usability of Intake24-NZ, nutrient data were not analyzed, and dietary feedback was not provided to the participants.

#### Usability Survey

Subsequently, participants used the second web link to complete the usability survey with questions on general experience and user-friendliness of the tool, searching and selecting food and drink items, estimating portion sizes, perceived usefulness of prompts, use of sandwich and salad builders, and support received. Parent proxies answered the survey questions related to their own experience of using Intake24-NZ on behalf of their child and were asked two additional questions: (1) rating the difficulty of reporting their child’s intake and (2) whether there were foods or drinks consumed by their child that they could not report or did not know the details of. For most quantitative questions, participants were given the option to clarify their answers using open text boxes. Several types of questions were used, such as single-answer questions, multiple-choice questions, Likert scales, and ratings.

### Statistical Analysis

Participant demographics and quantitative usability survey responses were analyzed descriptively, including percentages for categorical variables and mean (SD) for continuous variables. Analyses were conducted using Excel (version 2308; Microsoft Corporation). Subgroup analyses were not conducted.

Screen and voice recordings were analyzed qualitatively by extracting data directly relevant to the usability of Intake24-NZ. A total of 4 members of the research team (BF, CH, JG, and MM) were trained to identify and extract usability issues by recording the timing and actions of each participant. Subsequent thematic analysis of these usability issues involved identifying and analyzing patterns within the data to group recurring individual issues into themes and subthemes [18]. The thematic analysis was conducted by 3 researchers (BF, CH, and SM), who met regularly to discuss the findings and ensure consistency of interpretations.

At times when the observational data (participant actions) contradicted the participants’ survey answers, survey data were amended on the basis that the observational data were more reflective of the truth (eg, the participant indicated that they used the sandwich builder, while the screen recordings showed this was not the case). The results presented indicate where any adjustments were made.

## Results

### Participant Demographics

A total of 37 participants completed the dietary recall in Intake24-NZ and the usability survey. Participants represented a range of age groups, ethnic groups, and other key demographics, as outlined in detail in Table 1.

**Table 1:** Demographic characteristics of the participants completing a 24-hour dietary recall in Intake24-NZ (N=37).

Demographic characteristics		Values, n (%)
**Age group**		
	Parent proxies for children aged 2 to 10 y	8 (22)
	Children (11 to 15 y)	9 (24)
	Adults (16 to 64 y)	15 (41)
	Older adults (≥65 y)	5 (13)
**Gender**		
	Men and boys	9 (24)
	Women and girls	28 (76)
**Ethnicity**		
	Māori	11 (30)
	New Zealand European	18 (49)
	Pasifika	2 (5)
	Other^a^	6 (16)
**Highest qualification (n=28)^b^**		
	Secondary school	2 (7)
	Diploma, certificate, or trade	2 (7)
	Undergraduate degree or bachelor’s degree	7 (25)
	Postgraduate degree or master’s degree	17 (61)
**Background in nutrition or dietetics (n=28)^b^**		
	Yes	6 (21)
	No	22 (79)

^a^Other ethnicities included American, Australian, Dutch, Middle Eastern, Filipino, and Cambodian people.

^b^Excluding children.

### Usability Survey

#### Overview

Quantitative data are presented for the total sample (Table 2). No further subgroup analyses were undertaken due to the small number of participants within each age group. While some differences in scores by age were apparent, no conclusions can be drawn with any level of statistical certainty.

**Table 2:** Participants’ survey responses to assess the usability of Intake24-NZ (N=37).

Survey questions and answers				Values^a^
**General usability and accuracy**				
	**Intake24 was easy to follow and understand**			
		Score^b^, mean (SD; range)	3.8 (0.6; 2-5)	
		Agree or strongly agree, n (%)	31 (84)	
		Neutral, n (%)	4 (11)	
		Disagree or strongly disagree, n (%)	2 (5)	
	**I felt confident using Intake24**			
		Score^b^, mean (SD; range)	4.0 (0.8; 2-5)	
		Agree or strongly agree, n (%)	30 (81)	
		Neutral, n (%)	5 (14)	
		Disagree or strongly disagree, n (%)	2 (5)	
	**The information about my food and drinks that I recorded in Intake24 is the same as all the foods and drinks I had yesterday**			
		Score^b^, mean (SD; range)	4.6 (0.6; 3-5)	
		Agree or strongly agree, n (%)	35 (95)	
		Neutral, n (%)	2 (5)	
		Disagree or strongly disagree, n (%)	0 (0)	
**Finding foods and drinks**				
	**Did you have any problems finding the foods and drinks that you ate or drank in Intake24? n (%)**			
		Yes	24 (65)	
		No	13 (35)	
	**Did the foods and drinks listed in the search results match the items that you searched for? n (%)**			
		Yes	19 (51)	
		Sometimes	18 (49)	
		No	0 (0)	
**Portion size estimation**				
	**Did you have any problems with estimating the amount of food or drinks you had in Intake24? n (%)**			
		Yes	10 (27)	
		No	27 (73)	
	**Were there any foods or drinks that did not have your preferred way of describing the amount you had (eg, cups, tablespoons, butter on toast, or different size glasses)? n (%)**			
		Yes	8 (22)	
		No	29 (78)	
	**Were there any portion size photographs where you would have liked the food or drink sizes to be presented in a different way? n (%)**			
		Yes	6 (16)	
		No	31 (84)	
	**Were there any foods or drinks that you found particularly hard to estimate the amount of that you ate or drank? n (%)**			
		Yes	9 (24)	
		No	28 (76)	
**Associated food prompts**				
	**Was it useful to have a follow-up question to add another item for foods and drinks usually eaten together (eg, sugar in your tea or milk on your cereal)? n (%)^c^**			
		Yes	35 (95)	
		No	2 (5)	
	**When asked if you wanted to add another food or drink item (eg, butter on toast), did the suggested foods or drinks include options that made sense to you? n (%)**			
		Yes	*34 (92)^d^*	
		No	*2 (5)*	
**Sandwich and salad builder**				
	**Did you enter a sandwich or salad into Intake24? n (%)^c^**			
		Yes	15 (41)	
		No	22 (59)	
	**If yes, did you notice the option to create your own sandwich or salad in Intake24? n (%)^c,e^**			
		Yes	*5 (33)*	
		No	*9 (60)*	
	**If you noticed the option, did you use the sandwich or salad builder to record your food? n (%)^c,e^**			
		Yes	*5 (100)*	
	**If yes, how did you find building your own sandwich or salad?^e^**			
		Score^f^, mean (SD; range)	*4.2 (2.7; 2-9)*	
	**Were all the food items and fillings you wanted available (eg, sauces, spreads, meats, and vegetables)? n (%)^e^**			
		Yes	*4 (80)*	
		No	*1 (20)*	
	**If you did not notice the option, would you have chosen to build your own sandwich or salad if you were given the option? n (%)^e^**			
		Yes	*7 (78)*	
		No	*2 (22)*	

^a^Findings should be interpreted with caution, given that the total sample size is small.

^b^1=strongly disagree, 2=disagree, 3=neutral, 4=agree, and 5=strongly agree.

^c^Data amended based on observations (eg, the sandwich builder was not used, but participants indicated that they did use the sandwich builder).

^d^Values in italics indicate where data are missing because data were altered based on observational data, meaning that some values may not add up to 100%.

^e^Question was a part of skip logic and, therefore, not answered by all participants.

^f^Continuous range from 0 to 10; 0=very difficult and 10=very easy.

#### General Usability and Accuracy

Participants were generally positive regarding the ease of use (mean 3.8, SD 0.6; range 2-5), confidence in use (mean 4.0, SD 0.8; range 2-5), and accuracy of intake data collected using Intake24-NZ (mean 4.6, SD 0.6; range 3-5). Older adults tended to have lower average scores for the ease of use (mean 3.0, SD 1.0; range 2-4) and their confidence in use (mean 3.0, SD 0.7; range 2-4). Survey responses show that parent proxies found it easy to report their child’s dietary intake (mean 2.1, SD 1.0; range 0-3; 0=very easy to 10=very difficult). Only 2 (25%) out of 8 parents were “somewhat unsure” about the details of some foods and drinks their child consumed as these were eaten when the parent was not present (eg, at day care). Other parents did not report any issues specific to the proxy reporting.

#### Finding Foods and Drinks

Of 37 participants, 24 (65%) had problems with the search function in Intake24-NZ in relation to ≥1 foods and drinks they consumed, with children experiencing these problems the least. The key issues specified included some foods or drinks not appearing in the list of search results and the best possible matches not being listed at the top of the search results. Participants who reported that they could not always find an appropriate match (18/37, 49%) explained that the exact items or close matches were not listed in the search results, no homemade options were available to select, or essential food items were missing from mixed dishes (eg, lasagna not including vegetables). Most participants resolved this issue by selecting the closest match available, entering the mixed dish as separate ingredients, or reporting the food as missing from the food list. Missing foods were recorded by 13 (35%) of the 37 participants, whereas 9 (24%) participants said that they were not aware of the option to report missing foods or drinks.

#### Portion Size Estimation

Of the 37 participants, 10 (27%) reported problems with estimating the amount of food and drink they had consumed. Participants described difficulties in estimating amounts of some specific types of food (eg, meat), a preference for a different way of estimating their portion size (eg, in metric units [grams], “bite sizes,” or “splashes”), not being able to report smaller than typical portions (mainly reported by parent proxies), foods in the photos not matching the food item selected (eg, an image of a cucumber on a plate to represent the portion size of courgette), and being unsure about what a standard unit was (eg, the standard unit for spinach is *number of leaves* consumed (ie, 1 spinach leaf is 1 standard unit)). However, the size of the spinach leaves can vary, making it difficult for the user to know how to report the amount.

#### Associated Food Prompts

All participants were prompted about foods and drinks that are commonly consumed together. The majority of participants (34/37, 92%) indicated that the associated food prompts were useful for foods and drinks that are usually eaten together and that these questions made sense.

#### Sandwich and Salad Builder

Although 41% (15/37) of the participants entered either a salad or sandwich in Intake24-NZ, only 5 (33%) of those participants used the available builder to record the ingredients in their salad or sandwich. These participants scored the ease of use of these builders 4 out of 10 (SD 2.7; range 2-9; 0=very difficult to 10=very easy). Participants were unable to easily find their salad vegetables (ie, food categories within salad builder were hard to navigate), they did not know the exact ingredients, or experienced technical issues (eg, Intake24-NZ did not allow them to proceed to the next step in the builder). Of the 15 participants who entered a salad or sandwich, 9 (60%) were not aware of the builder option, of which 7 (78%) participants said that they would have used it had they known the option was available.

#### Suggested Improvements and Key Features

When asked for suggestions for improvements to Intake24-NZ, participants highlighted that the search results were difficult to navigate, mainly due to the order in which results appeared, the number of items displayed, and the inclusion of irrelevant foods. In addition, participants provided suggestions to improve portion size estimation by adding new food images, allowing reporting of smaller portions, including the option to record weight in grams, and providing clearer instructions. Nevertheless, the portion size estimation feature, including portion images and multiple size options, was perceived as one of the best features of the tool, as it provided a visual aid to estimate portion size consumed and was perceived to increase the accuracy of the reported portion and easy to use. In general, participants liked the stepwise process of reporting their intake and thought the tool was easy to navigate.

#### Previous Experience, Instructions, and Support

A total of 14 (38%) of the 37 study participants reported having previously used a similar web-based or mobile app to record their food intake. Approximately half of the participants (19/37, 51%) reported having watched the instruction video before completing their recall, while 10 (27%) did not watch it and 8 (22%) only partly watched it. Feedback on the instruction video from those who (partly) watched it included suggestions to increase the narrator’s voice volume and use a narrator with a local accent, improve visibility (eg, making the text and visuals larger), and add new content. Just less than one-third of the participants (12/37, 32%) needed support from someone else while using Intake24-NZ, with most help provided to children and older adults. Support needs included requiring help from family members, friends, and the research team to navigate the tool, estimate portion sizes, use the search function to find foods, and solve technical problems.

### Usability Observations

#### Overview

A total of 26 screen and voice recordings were collected during dietary recall completion. Owing to technical or user difficulties, neither screen nor voice was recorded for some recalls (6/37, 16%), only audio recordings were obtained for some recalls (3/37, 8%), and only screens were recorded for the remaining recalls (2/37, 5%).

From the observations, 10 main usability themes were derived: problems relating to search terms used, search results, meal menu, food categories, portion size estimation, associated food prompts, salad and sandwich builders, missing foods function, support, and issues not otherwise classified. Findings are summarized below and in Multimedia Appendix 2, with examples given for issues identified. The most commonly observed problems were with search terms used, search results, portion size estimation, and associated food prompts.

#### Search Terms

Contrary to the instructions provided in the introductory video and written guidance, participants often provided too much detail when entering foods or drinks into Intake24-NZ, such as the amount of food consumed and brand names, or they added multiple food items on 1 line. In case of the latter, the search function recognized multiple items and prompted the participant to separate them. Occasionally, this prompt also appeared when not appropriate, causing confusion and leading to some participants incorrectly separating a single food or drink item into multiple individual items when searching. Participants were not always able to undo these changes due to issues related to the meal menu (Multimedia Appendix 2). In addition, observations showed that typing errors or spelling mistakes had further adverse consequences for the accuracy of search results (Table 3).

**Table 3:** Usability observations related to search terms used when completing a 24-hour dietary recall in Intake24-NZ.

Observations	Further details and examples
Participants entered multiple food or drink items in the search bar.	A participant entered, “Yogurt, seeds, nuts, dried fruit, blue berries, olive oil, cinnamon” as 1 string into the search bar.
The search function prompted participants to separate food or drink items, which was not always appropriate.	A participant entered “Up and Go,” the brand of a breakfast drink, and the tool asked to split this drink item into “Up” and “Go.”
Search terms included excessive detail, such as the amount of the food, number of food items, or cooking method.	A participant entered, “Half a pouch of mushroom soup Naked Locals brand 250 gms.” A participant entered, “One and a half cups of oat cereal.”
Participants searched for foods or drinks using specific terms or brands, while food list items are mainly described using generic terms.	A participant searched for “doritos,” while this food is described as “corn chips” in the food list.
Participants made small spelling or typing errors, and special characters were often not used.	A participant searched for “malteesers” instead of Maltesers, and the system returned many irrelevant search results such as multigrain breads. A participant searched for “kumara” without a macron. Kūmara (sweet potato) is spelled with a macron in the food list, and therefore, no relevant search results were displayed.

#### Search Results

Although relevant foods were mostly available and participants were usually able to find their foods and drinks, several issues with the search results were identified (Table 4). Errors with the format of search terms entered caused the search engine to list food and drinks that were not relevant or not identify any food and drink items. Other observed reasons for suboptimal search results included that the search algorithm did not deal well with minor typing errors, the order of the results shown was not intuitive, some items were not available in the food list, or not all possible matches were shown due to the page limit (ie, ≤30 items could be displayed at a time). This led to participants using different strategies to overcome these issues, such as changing their search terms, using food categories (Multimedia Appendix 2), or reporting a food as missing. Consequently, it took much more time (eg, having to undertake additional steps or to thoroughly review the listed food items) to navigate the search results and to find the foods and drinks the participants were looking for, with some participants expressing frustration. At times, these issues led to the selection of the wrong item.

**Table 4:** Usability observations related to search results when completing a 24-hour dietary recall in Intake24-NZ.

Observations	Further details and examples
No relevant search results were listed due to the search terms used.	A participant searched for “1 and a half cups of cereals (oats and muesli),” and the search results returned the following single option: Toddler/kids cereal bar eg, Rafferty’s Garden oat bars, Heinz muesli fingers.
Many irrelevant search results were listed.	A participant searched for “apple slices,” and the results listed were both a variety of apples and sweet slices (eg, caramel slice).
Food and drink items did not appear in the list of search results for unknown reasons.	A participant searched for “cornbeef,” and the food list items of *corned beef, canned* and *corned beef, not canned* did not appear in the search results.
The sequence of the search results was not intuitive.	Most popular items were not listed at the top of the search results. A participant searched for “apple” as they consumed a raw apple, and the best possible match could only be found halfway down a list of 30 apple-related food and drink items.
Food and drink items were not listed in the search results, as they did not exist in the food list.	Some participants searched for a lemon, honey, and ginger drink that was not available in the food list.
Not all potentially relevant search results were shown due to page limit.	Many participants searched for “milk,” and the search results did not include the most commonly consumed types of milk. The most likely reason is that there are many different types of milk and other food descriptions that also include this term (eg, milkshake and hot chocolate made with cow’s milk). The large number of search results did not fit within the page limit.
Participants were sometimes unsure or unable to match consumed food to an item in the food list even though an appropriate match was available.	A participant searched for “peanut brownie,” and *brownie* was listed at the top of the search results and was the only type of brownie available in the food list. Instead of matching their peanut brownie to the food list brownie, the participant reported it as a missing food.

#### Portion Size Estimation

Generally, participants liked the portion size images and thought they were useful aids to estimate the amount of food eaten. Suggestions were made to change some existing images or create new ones to improve portion estimation. Moreover, issues were observed that could have caused difficulties with reporting portion size (Table 5). Some participants were confused by the portion size images, as several images display similar foods (shape, size) but not the same food as the participant consumed (type). For example, images of cucumbers were used to estimate the portion size of courgettes. This could have led to lower user satisfaction and misreporting of portion sizes. Observations also indicated that participants may have been confused by some guide images and selected the image that best matched the type of food or drink eaten rather than the amount consumed. There was an option to use a standard portion (eg, measuring cups) to estimate portion size, but most participants did not know what the term *standard portion* meant, which resulted in them selecting this estimation method less often. Furthermore, participants commented that some portion sizes were either too big or too small and they could not adjust the displayed portions to the correct size. Once the portion size step was completed, the system did not allow participants to amend their reported food quantities. Issues inherent to the 24-hour recall method were also observed, with some participants unable to remember how much they had eaten and match it to the portion size images provided.

**Table 5:** Usability observations related to portion size estimation when completing a 24-hour dietary recall in Intake24-NZ.

Observations	Further details and examples
Some participants were confused when a portion size image displayed a food that was different from what they had selected.	A participant commented, “None of these are the Peckish [brand name] crackers” and “The images do not really match the description and I do not think any of these are rice crackers, these are all grain crackers,” when viewing the guide image displaying different types and sizes of crackers. The guide image did include a cracker of the same shape and size as Peckish rice crackers, which the participant could have selected.
Participants sometimes selected the best match from the guide images for the type of food or drink eaten rather than the amount consumed.	A participant had canned spaghetti and was confused about the image of different can sizes that did not include a can of spaghetti. They reread the instructions saying, “select the item you had or the closest match to...” and ended up selecting the can of minestrone, explaining this to be the closest match as it has tomato sauce with pasta in it.
Portion size estimation was difficult for some foods using the existing portion size estimation options, with several participants suggesting portion size measures or images be added or amended.	A participant only had the option to report the amount of Brussels sprouts by the number of individual sprouts. They suggested having an as-served image of Brussels sprouts on a plate. Participants wanted to report the exact amount consumed using metric units (eg, weight or volume).
Some participants did not understand how to select or use the portion size estimation options.	A participant wondered what standard portion meant. They decided to select the guide image picturing different-sized glasses because they did not know what the standard portion was. Participants have to click the standard portion button first to then see what unit (eg, tablespoon; cup; and small, medium, and large [food]) can be used to estimate.
Participants reported some portion sizes being too small, while others commented they were not able to decrease the size enough.	A participant wanted to report one-third of an apple, but fractions only go down to half, which was the closest amount they could report.
Participants were unable to change the portion size selected after completing this step.	A participant reported having 3 whole large water bottles when asked, “Please choose how many of tap water you had.” The total volume of water added up to an unrealistically high amount of 7500 mL. The participant noticed that they had reported too much water. When trying to change this, the system responded, “We have all the information that we need regarding your tap water at this time.”

#### Associated Food Prompts

All participants were prompted about foods commonly consumed together, with several participants adding an associated food or drink item following the prompt. The Intake24-NZ tool is programmed to recognize the foods or drinks previously entered by the participant and to display an option to say, “I have already entered this,” with the prompt question. However, this option did not always appear, causing some participants to report the same food twice. In turn, difficulties related to the meal menu (ie, unsure how to remove foods and drinks once entered; Multimedia Appendix 2) prevented participants from removing such duplicates. The timing and wording of the prompts were also perceived as problematic as participants were sometimes unsure which foods and drinks the prompts related to, leading to misreporting, frustration, and potentially longer recall completion times. Examples are provided in Table 6.

**Table 6:** Usability observations related to prompts when completing a 24-hour dietary recall in Intake24-NZ.

Observations	Further details and examples
Several prompts did not make sense or were irrelevant to NZ^a^ users.	Participants were prompted about whether they had garlic bread with their macaroni cheese and beef lasagna. This meal combination is not common in NZ.
Prompts often worked well to remind participants about items commonly consumed together.	A participant remembered to add soy sauce to their dumplings when prompted.
Participants were unsure which prompts related to which food or drink item, as the timing and wording of some prompts were confusing.	A participant completed the portion size steps for all their lunch foods, including a salad and smoothie. After completing the portion size estimation for their smoothie, a prompt was displayed asking about their salad (eg, “How many people did this Salad serve?”), and they were required to enter a recipe name for the salad. The participant was confused as they just reported their smoothie and typed “this was supposed to be smoothie” as the recipe name.
At times, the *already entered* option did not appear when asked about adding foods that are commonly consumed together.	A participant recorded ice cream and fruit salad. They were prompted about having had ice cream with their fruit salad. The only logical option for the participant to select was “Yes, I had some,” following which they recorded their ice cream again.

^a^NZ: New Zealand.

## Discussion

### Principal Findings

This study evaluated the usability and functionality of Intake24-NZ, a web-based 24-hour recall dietary assessment tool. Usability testing is an important aspect of tool development, given its potential to identify the causes and consequences of usability issues. We used a unique and comprehensive suite of methods, incorporating findings from a usability survey, think-aloud recordings, and recorded observations to understand the user experience of Intake24-NZ. The latter, in particular, provided rich insights that do not rely on the users’ capability to identify, recall, and report problems [9]. The survey findings showed that although most participants said they found the tool easy to use and felt confident using it, some had difficulties finding their food and drinks in the food list and in estimating portion sizes. While the observational data supported the survey findings, they also identified some additional issues and potential underlying causes and consequences. Additional issues from the observations related to the search terms, search results, and prompts associated with specific foods. The identified issues could significantly impact the accuracy and quality of the recorded dietary intake data unless corrected.

Usability testing of a similar version of Intake24 was conducted with Scottish young people and adults (aged ≥11 years) who completed a feedback questionnaire after self-completing up to 4 recalls (n=182, 74% of the total sample) [19,20]. Findings indicated that 83% of the participants found Intake24 easy to follow and understand, 81% felt confident using the tool, and 78% thought the tool accurately captured their intake [19]. Following this initial study, a similar but larger study was conducted in 2018, with approximately 815 participants (aged ≥11 years) self-completing 2 recalls and a feedback questionnaire [15]. Most participants (88%) agreed that Intake24 was easy to use, 81% felt confident using it, and 71% believed that their intake captured in Intake24 accurately reflected what they consumed. Findings from both studies are generally comparable to our survey results, which found that 84% (31/37), 81% (30/37), and 95% (35/37) of the participants agreed with those same statements.

Despite these high levels of agreement and other positive comments about overall experiences with Intake24-NZ, some problematic issues were identified. Approximately two-thirds (24/37, 65%) of our participants reported problems in finding foods and drinks in the Intake24-NZ food list. On the basis of the observed data, these issues likely originated from the incorrect use of the search bar such as including multiple items on 1 line, adding too much detail (eg, the amount or specific brands), or small spelling mistakes. Some of these issues potentially could have been avoided as participant instructions were to use generic search terms and separate each food and drink, but the instructions were often skipped, or the video was only partly watched. This unfortunately is a common behavior among software users [21]. The search term issues subsequently impacted the search results adversely, with either limited relevant food items displayed or many irrelevant items showing. These findings are not unique to Intake24-NZ, as such search-related problems have been identified with similar dietary recall tools. Two usability studies of the Automated Self-Administered 24-hour tool also reported that participants entered multiple items and detailed descriptions in the search bar, thereby not returning the desired search results and being unable to find relevant food items [9,22]. A more recent study compared university students’ perceived problems between Intake24 (Australian tool; version 4) and the Automated Self-Administered 24-hour tool using think-aloud methods [23]. For both tools, participants experienced problems in knowing what to search for and how specific to be.

The long search result list and the nonintuitive ordering of displayed foods are problematic, given that users tend to select items that are listed at or near the top of a list [24]. Kirkpatrick et al [22] concluded that their participants particularly struggled to find the foods they consumed when there was a long list of search results. Frustration because of these issues may further impair the accuracy of data collected by clouding participants’ memory [23] and making them less likely to complete a recall again [9]. Therefore, these usability issues could decrease the accuracy of dietary data collected and negatively impact studies or national nutrition surveys that require multiple recalls to be completed.

The inability to find foods in Intake24-NZ originates from both user errors and system issues, each needing different solutions. Since this usability testing was conducted, an updated version of Intake24 (version 4) has been released by the University of Cambridge, addressing several of the identified usability issues. The search function and ordering of search results have been updated using frequency data that identified the most commonly selected items. This will improve the type and order of foods displayed to the participants, listing those most frequently selected items at the top of the list. Issues finding a food item have potentially been further minimized by removing the page limit, adding new foods to the database based on those identified through the missing foods function, and updating the food list to reflect recent changes in the food supply. Where possible, food names have been reviewed to align with commonly used search terms. Furthermore, an improved and engaging instruction video has been developed to better guide the participants on how to search for foods and drinks more effectively.

The images in the portion size estimation step were well liked, with several participants commenting that these provided a useful visual aid that positively impacted the accuracy of the reported amounts eaten. These comments align with previous research concluding that image-based portion size estimation aids facilitate accurate dietary intake reporting [25,26]. In line with our survey findings, other Intake24 usability studies found that most participants (93% [13] and 91% [15]) did not report portion size estimation issues. However, neither of these earlier studies collected observational data. Our screen and voice recordings revealed that participants experienced issues that they either were not aware of or may not have perceived as a problem themselves and were, therefore, not captured in the survey. For example, some participants were unaware that the portion size estimation step involved selecting the image that best reflects the amount of food eaten rather than the type of food. Some issues specific to portion size estimation (eg, unable to further decrease the size, unable to change portion size once reported, or no available images) prevented participants from accurately reporting their portions. This, on top of the fact that portion size estimation is a difficult task by itself [27-29], leads to measurement error that significantly impacts the validity of dietary data. Therefore, addressing these observed issues is important, and the most recent upgrade to Intake24-NZ version 4 included several major modifications that are assumed to help participants with portion size estimation, one of which is the ability to adjust the portion size that is now clearly indicated in the meal menu using an *edit* button. This button allows participants to either change the food, edit the portion size, or delete the food for any of the already reported food or drink items. More generally, clearer instructions on the purpose of the portion size step and how to use the estimation options have been included in the instruction video and the written information (ie, “Please select the item you had or the closest match to [food item]” has been changed to “Select the image that is the closest match to the size of [food item] you had”). The portion size image directory available in Intake24-NZ was also expanded (ie, new portion size images were taken), and portion size images already assigned to food list items were reviewed. Given the confusion around the meaning of a standard portion, the types of standard portion options available have been listed together with all other portion size images (ie, the participant no longer has to click on the standard portion button first to be able to see which units are available for selection).

Associated food prompts helped participants remember to report additional items, which may improve the accuracy of a dietary recall and reduce underreporting. However, the wording and timing of the prompts were confusing to some participants and sometimes led to duplicate entries. Clearer instructions on how to amend or remove foods entered have been added to the instruction video, but this feature has likely also become more intuitive with the addition of the edit button. In line with this, more flexibility has been provided to adjust the recall entries at the meal level, meaning that participants can now easily change or add foods, change the time of the meal, or delete the entire meal by simply clicking on the edit button next to the meal name. More significant amendments have been made to the tool with regard to the problems with duplicate entries as a result of the unclear answer options to the associated food prompts (ie, there was no option to say, “I have already entered this”). Changes to the process of answering associated food prompts include clearer guidance on which answer option to select (ie, “Choose ‘Yes, I had some’ if you have already entered it”), a list of the food items that the system recognizes as already entered, and a search bar to instantly look up and add associated foods not entered yet. After reviewing the associated food prompts, minor changes were made to clarify the wording (eg, “...to your [food item]”), aiming to help the participant better identify which food the prompt refers to.

Similar to any dietary assessment method, web-based 24-hour dietary recalls have limitations. Particularly when self-completed, additional challenges may be experienced or measurement errors introduced compared to when the recall is completed with assistance from an interviewer. In the context of a national nutrition survey that includes multiple dietary recalls, a first recall that is led or assisted by the interviewer could help participants familiarize themselves with the tool and recall process to be able to self-complete a second recall more efficiently and effectively. Nevertheless, there is a growing move toward self-completion of dietary assessments in surveys and research studies [2], and thus, it is important that improvements are made to Intake24-NZ to facilitate self-completion of recalls and future-proof it as a dietary assessment tool. Several recent reviews suggest that artificial intelligence (AI) may have a place in improving and advancing self-completed dietary assessment methods [30-33]. However, this is a relatively new research field in which the exact role of AI in web-based 24-hour dietary recall tools, and particularly its impact on usability, is not well explored, and concerns regarding accuracy, privacy, and integrity also remain unsolved [31]. It is important to continue exploring innovative AI applications to ascertain their potential to increase the usability and accuracy of these web-based tools and ultimately to contribute to more effective nutrition and public health research.

### Limitations and Strengths

Some limitations of this research should be noted. In this study, we deviated slightly from traditional 24-hour dietary recall methods, as participants were asked to think aloud, were made aware in advance of the dietary recall date, and were permitted to take notes or photos to record their food intake before the recall. While these methodological changes enabled us to focus on the usability of the tool rather than the recall process, they could have changed participants’ behavior when using Intake24-NZ in relation to search terms used, navigation patterns, and recall completion time, thereby perhaps not always reflecting usability issues encountered in a “real” recall scenario [9]. For example, participants’ increased awareness and prerecorded notes may have resulted in participants using more detailed search terms. In addition, a relatively small convenience sample was recruited, which limited our ability to compare findings across different age and demographic groups. Furthermore, participants were primarily recruited from the researchers’ network, which likely introduced selection bias. Our sample predominantly consisted of highly educated women, many with an interest in nutrition and health. This select demographic representation should be considered when interpreting the results. It is recommended that future evaluations of Intake24-NZ include a more diverse sample in terms of gender, education, ethnicity, and income level to enhance the representativeness of the findings. Finally, our evaluation of Intake24-NZ solely focused on its usability and did not include an assessment of its accuracy in capturing dietary intake data. Therefore, the findings cannot address the tool’s effectiveness in providing valid dietary intake data, and future research is recommended to compare Intake24-NZ to other, preferably objective, measures of dietary intake.

A notable strength of our study was the use of a mixed methods approach, which facilitated the collection of both quantitative and qualitative data. This comprehensive approach allowed for an in-depth understanding of usability issues associated with the Intake24-NZ tool, enabling us to identify areas for improvement and guide further development effectively. Despite being a small sample, our study, nevertheless, included participants from a range of age groups, a reasonable number of Māori participants, and various types of recalls (parent proxy vs self-completed). This ensured that we captured a diverse range of perspectives and experiences, enhancing the utility and applicability of the findings. In addition, the detail and depth of the data collected for the qualitative component of the study allowed us to discern both prevalent and infrequent issues. By integrating observations alongside participant survey responses, we also illustrated differences in findings, highlighting the importance of observational data in complementing self-reported feedback and facilitating the identification of areas for improvement. The screen recordings also helped to identify usability issues where participants encountered difficulties articulating their thought processes while completing a new task.

### Conclusions

Our research findings provide valuable insights into the usability of Intake24-NZ, and web-based 24-hour dietary recall tools more broadly, and contribute to a better understanding of factors that underpin measurement error in this type of dietary assessment. Although easy to use, both survey findings and observations highlight the need for further development to optimize the functionality of Intake24-NZ and thus enhance the accuracy of dietary intake data collected using this tool. Key usability issues related to finding the correct food and drink items in Intake24-NZ, portion size estimation, and use of associated food prompts. Our findings may be applicable to other 24-hour dietary recall tools and are important for researchers and developers designing or improving the functionalities of such tools. As such, usability testing should be undertaken as a matter of course during the development or modification of 24-hour dietary recall tools. Since this usability testing was conducted, version 4 of Intake24 has been released. Some of the identified issues have been mitigated in this new version, while others might have been introduced, meaning that further testing is required. Future usability studies involving Intake24-NZ should also assess the quality of the dietary intake data collected by comparing it to objective measures.

## Appendix

Multimedia Appendix 1 Overview of the multiple-pass dietary recall steps and functionalities in Intake24-NZ.

Multimedia Appendix 2 Usability observations related to other Intake24-NZ features, including the meal menu, food categories, salad and sandwich builder, missing foods function, support, and issues not otherwise classified.

## Abbreviations

AI: artificial intelligence
NZ: New Zealand

## Data Availability

The datasets generated and analyzed during this study are not publicly available due to ethical considerations but are available from the corresponding author on reasonable request.
