# Supplementary material for: Evaluating User Experiences and Preferred Features of a Web-Based 24-Hour Dietary Assessment Tool: Usability Study
Source: JMIR Form Res. 2024 Oct 18;8:e63823. doi: 10.2196/63823 (PMC11530719; doi:10.2196/63823)
Supplement: Multimedia Appendix 1 [file formative_v8i1e63823_app1.pdf]

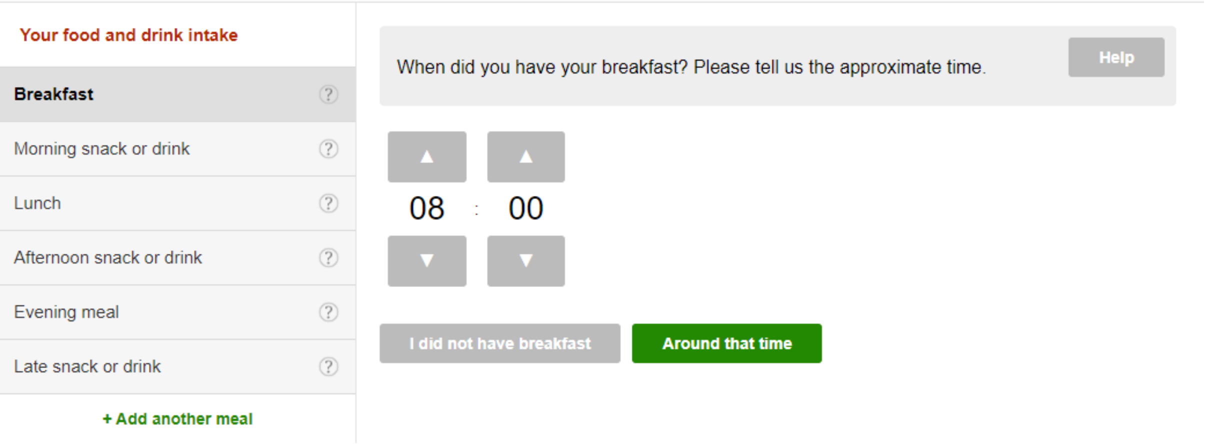

Figure S1. Selection of the time of a meal, snack, or drink.

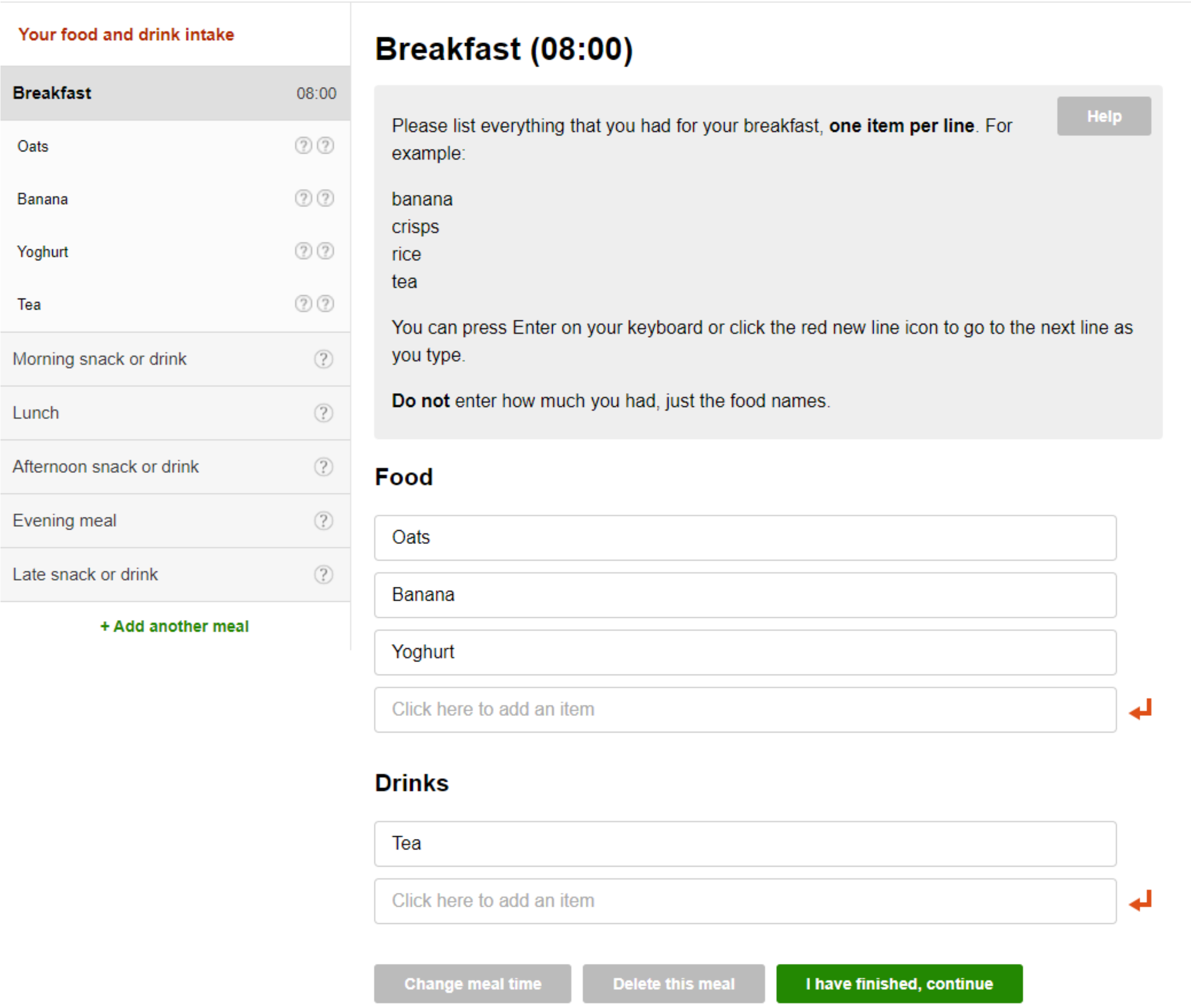

Figure S2. Entering food and drinks consumed for each meal or snack using free text.

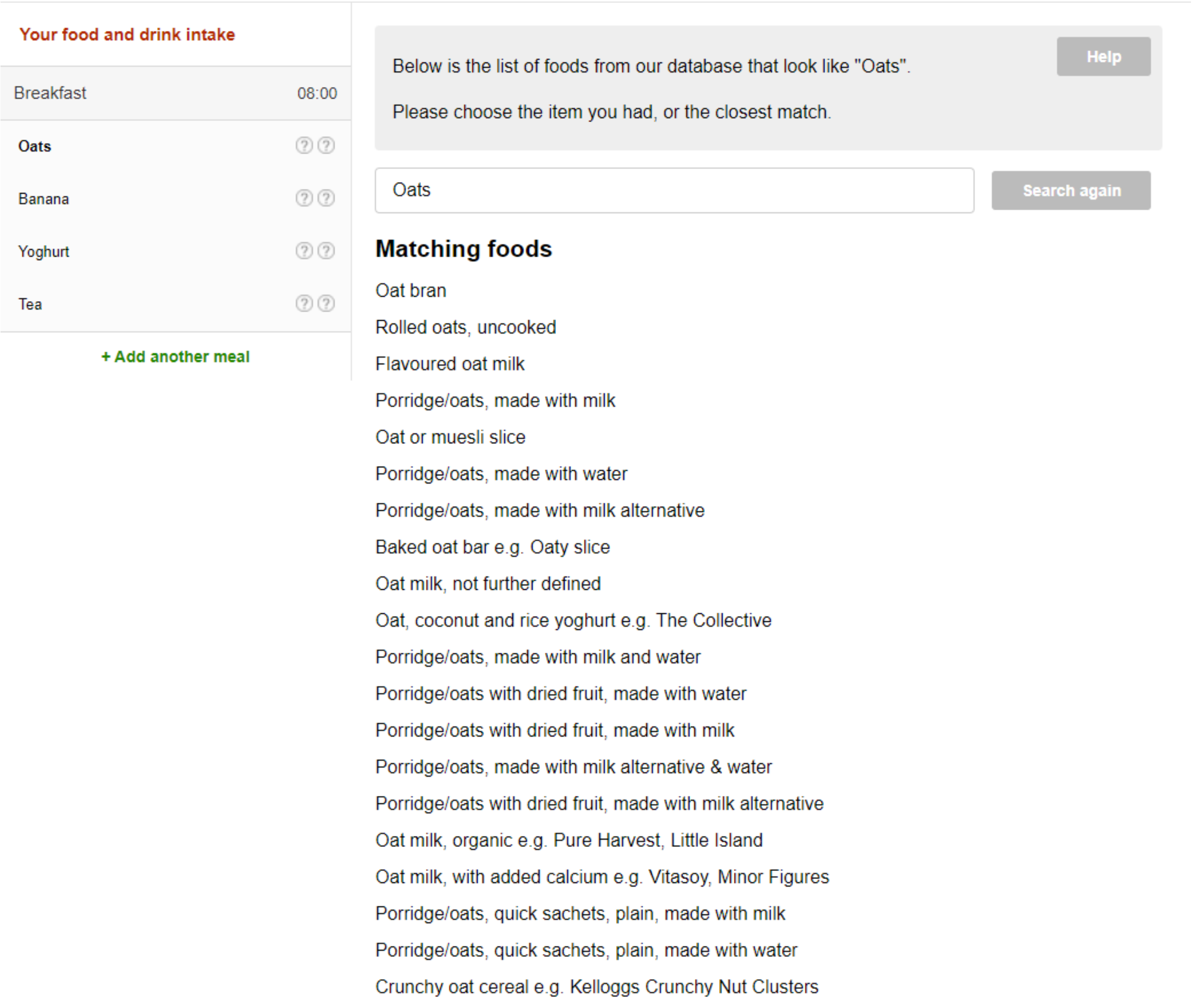

Figure S3. Search results listed from which to select the closest match.

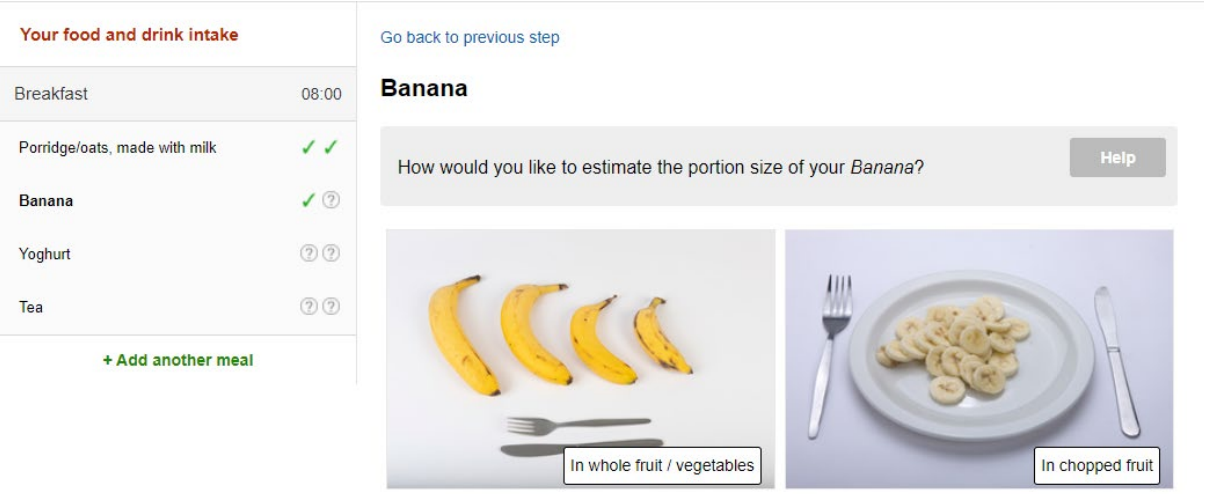

Figure S4. Portion size estimation options are provided to select the preferred estimation method (left: guide image; right: as-served image).

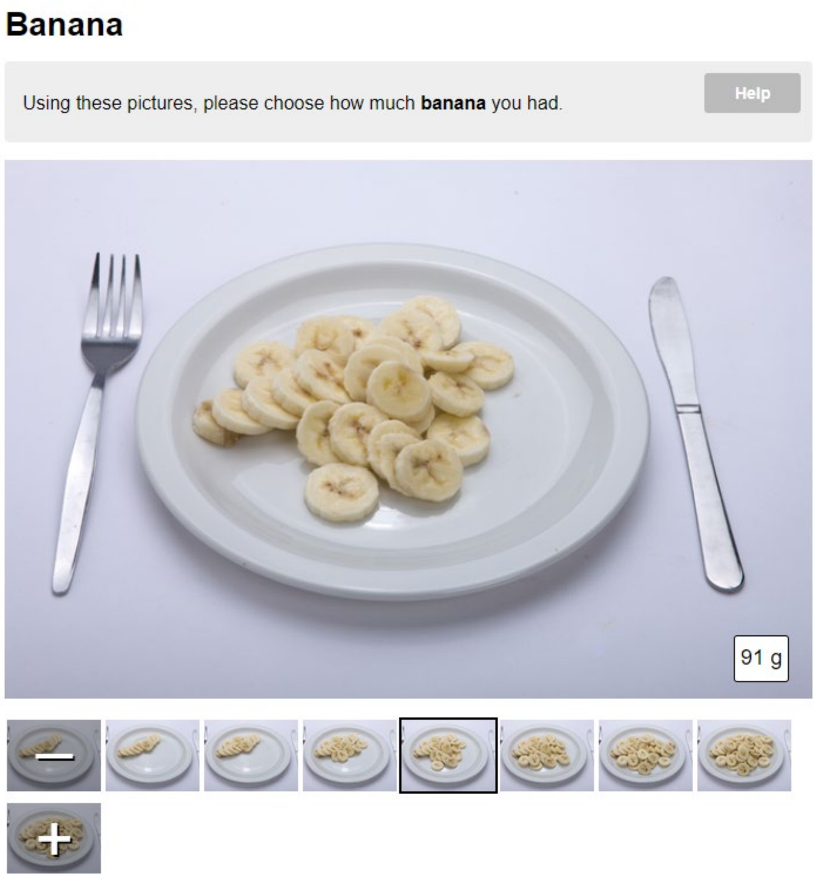

Figure S5. Portion size estimation using an as-served image of chopped bananas.

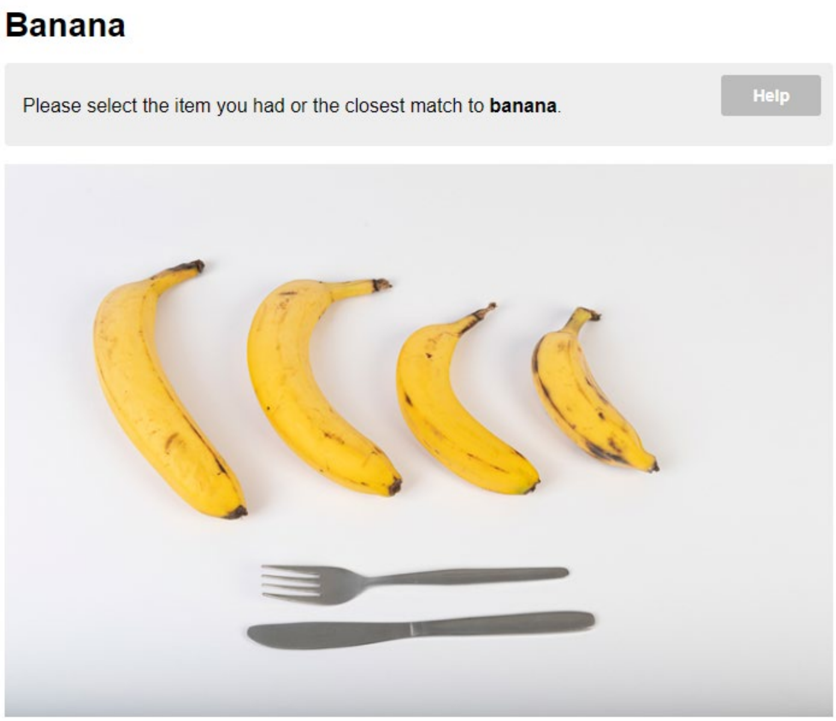

Figure S6. Step 1 of portion size estimation using a guide image of whole bananas; selecting the size.

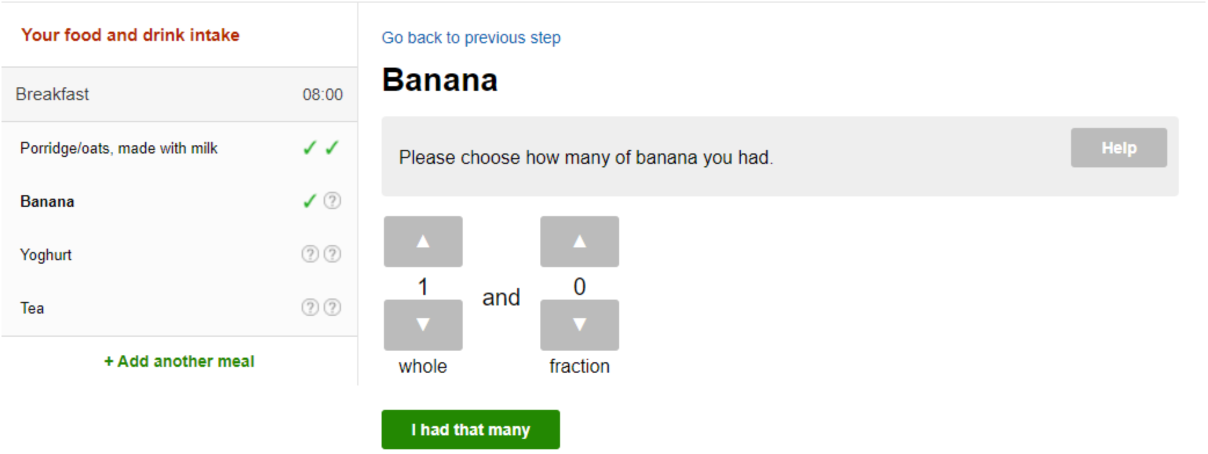

Figure S7. Step 2 of portion size estimation using a guide image of whole bananas; selecting the number of bananas consumed.

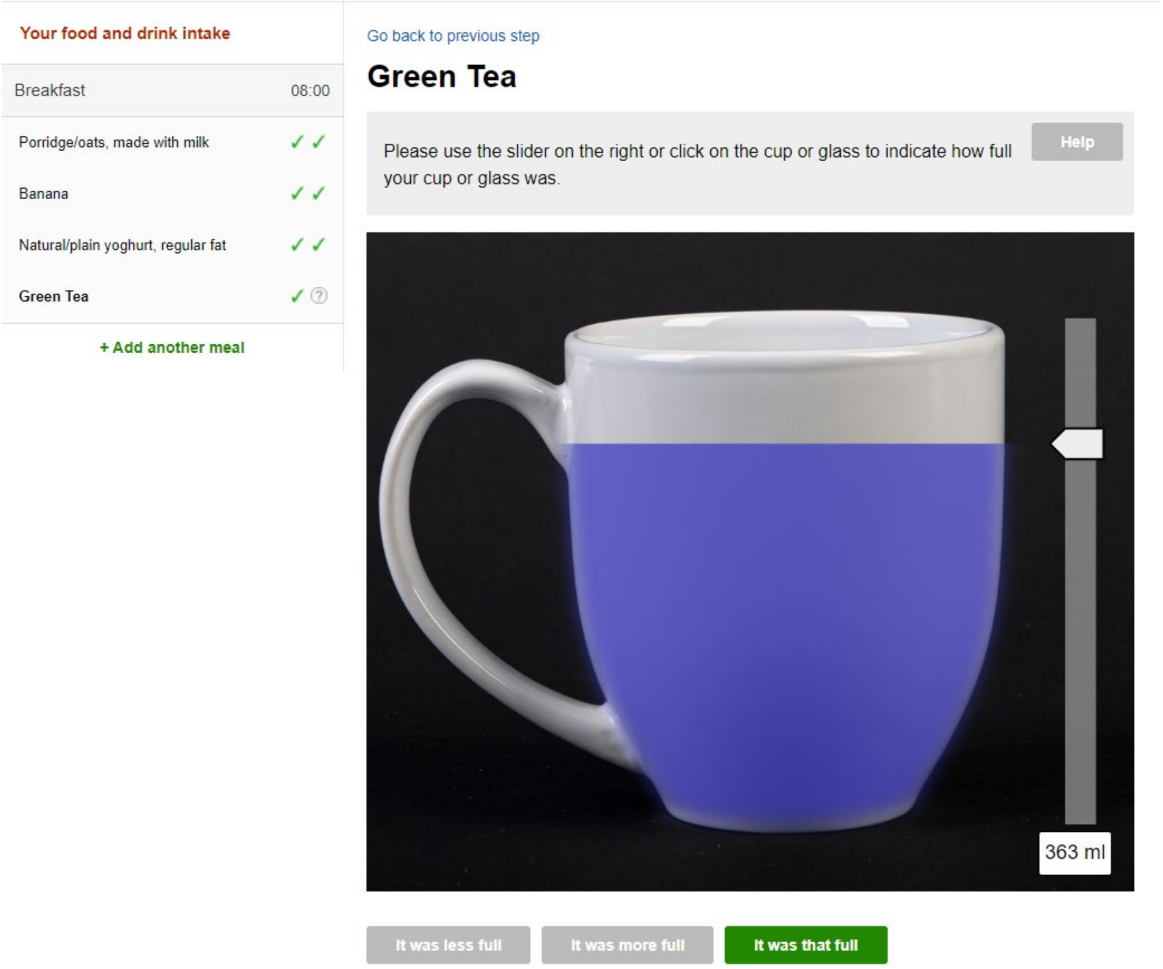

Figure S8. Portion size estimation of drinks using a slider.

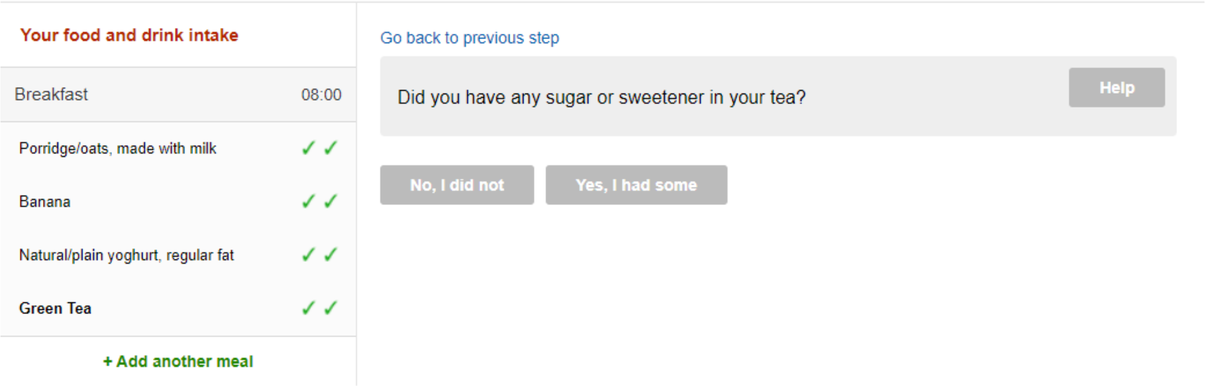

Figure S9. Associated food prompts ensure foods commonly consumed together are not forgotten (eg, sugar or sweeteners added to tea).

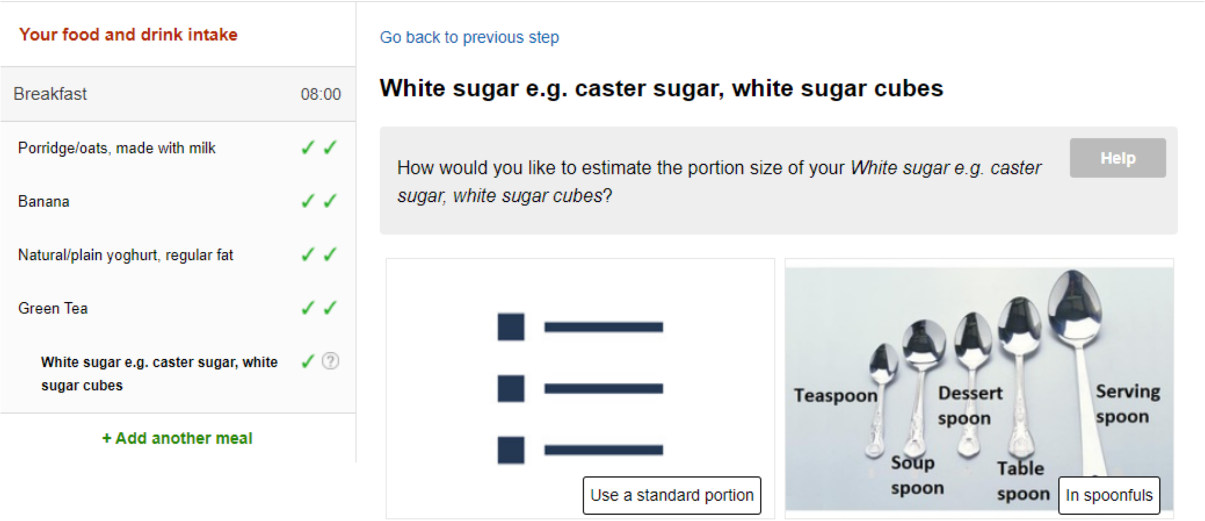

Figure S10. Similar steps are followed to record the portion size of the associated food (eg, sugar). Portion size estimation options are provided to select the preferred estimation method (left: standard portion; right: guide image).

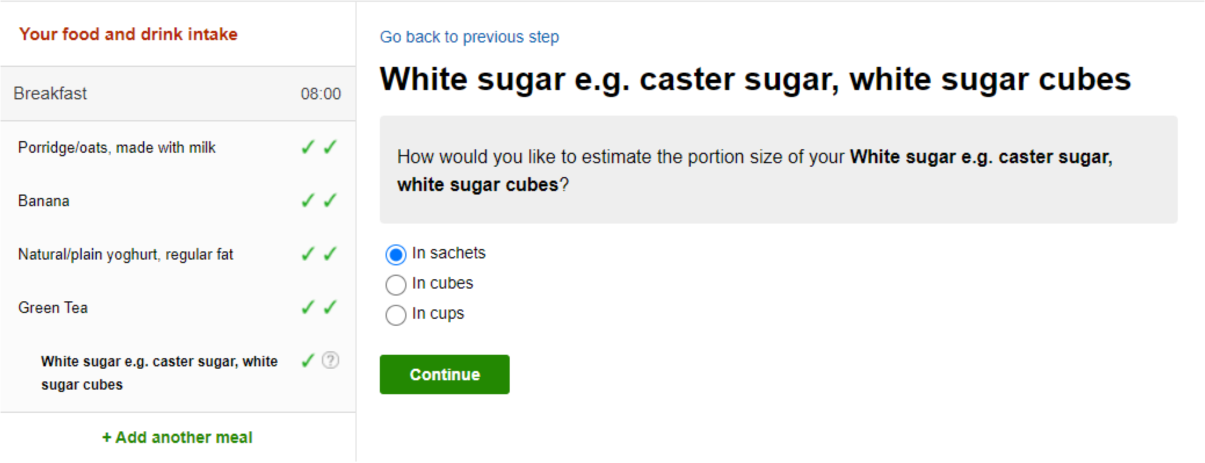

Figure S11. Portion size estimation using standard units (eg, sachets, sugar cubes, or measuring cups).

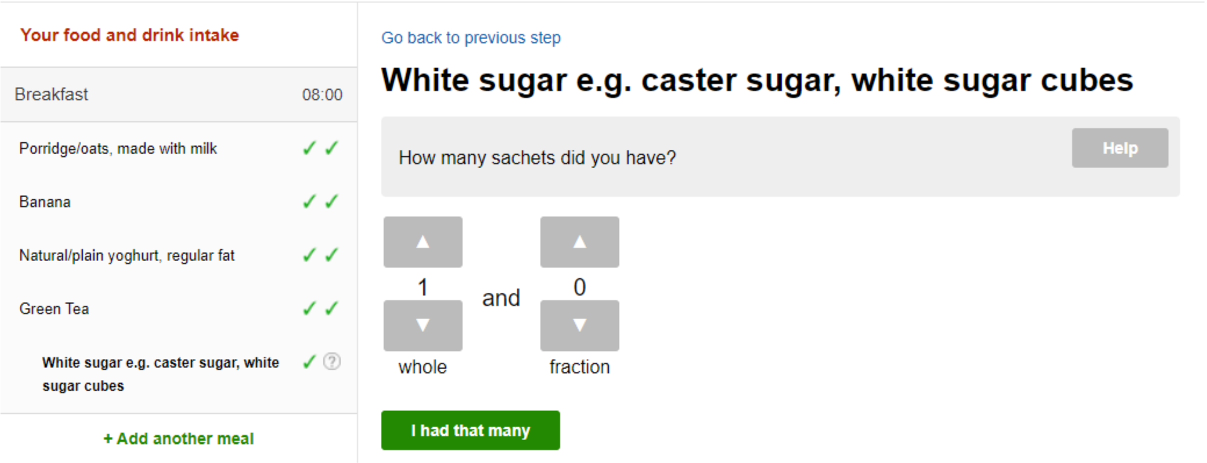

Figure S12. Portion size estimation using a sugar sachet (selecting the number of sachets added to the tea).

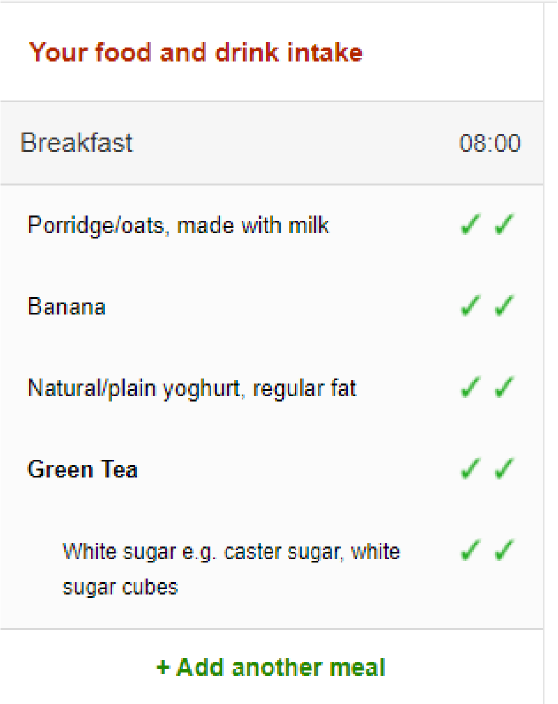

Figure S13. Meal menu providing an overview of the recorded meals and snacks including the food and drink items for each. This overview helps participants navigate and check the progress of the dietary recall process and allows for adding a meal or changing the meals or foods already entered.

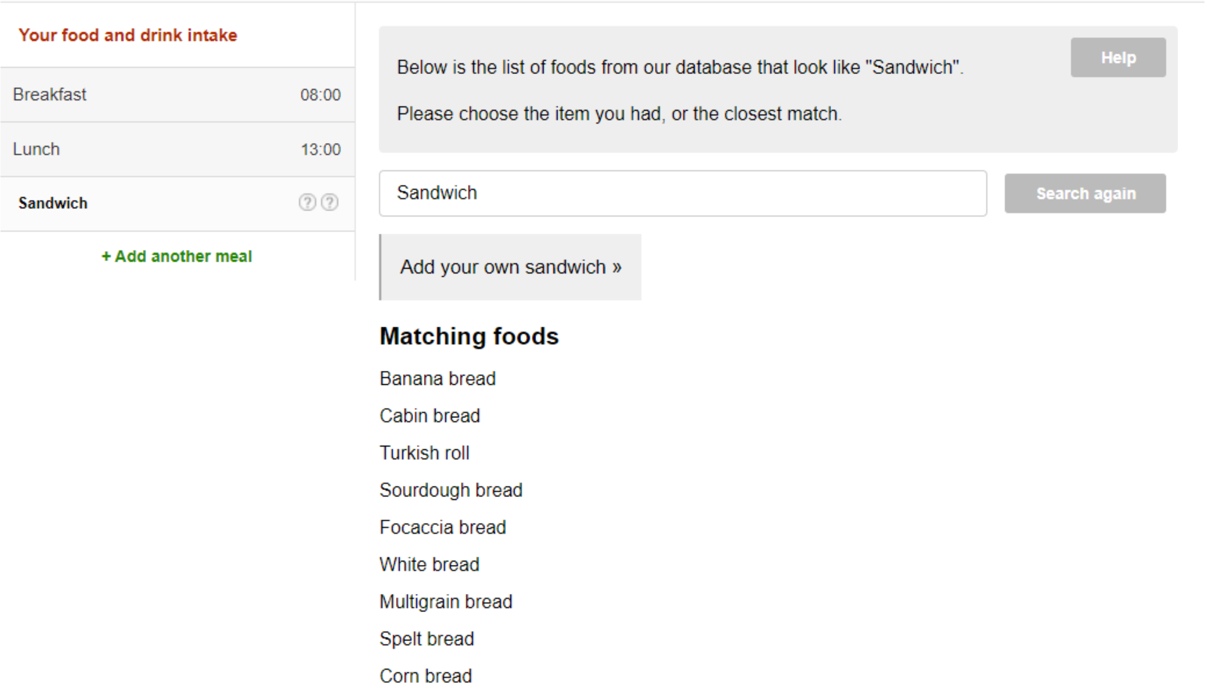

Figure S14. The sandwich builder option is provided to record all ingredients, including the bread, spreads, fillings/toppings (eg, cheese or spinach leaves), and sauces.

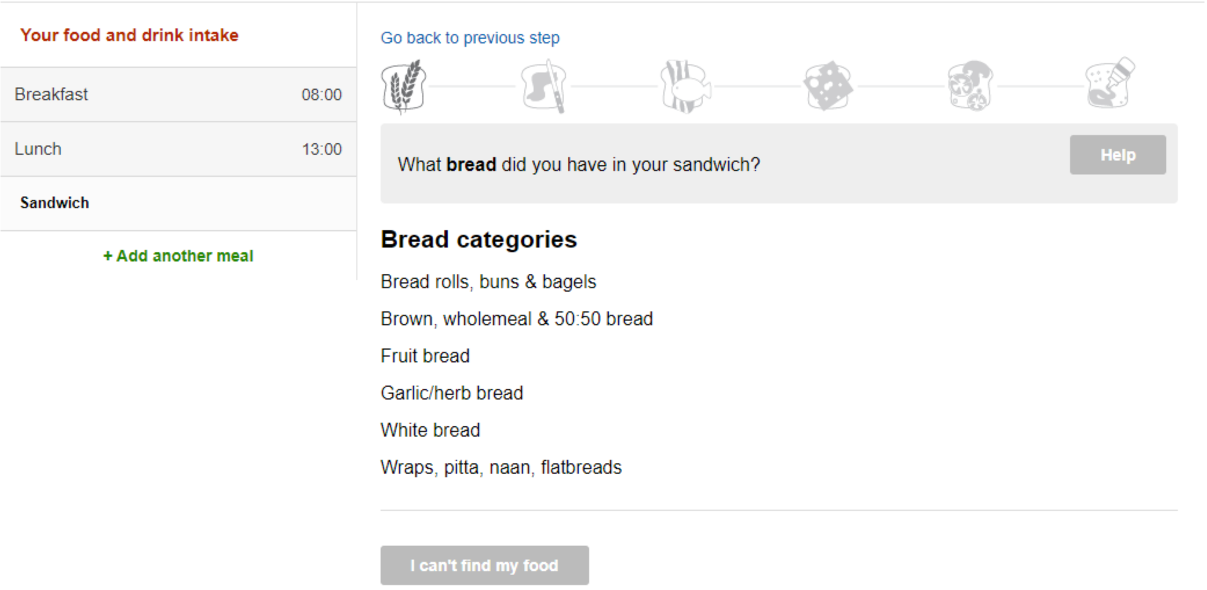

Figure S15. Step-by-step process to record all sandwich ingredients and their portion sizes using the sandwich builder.

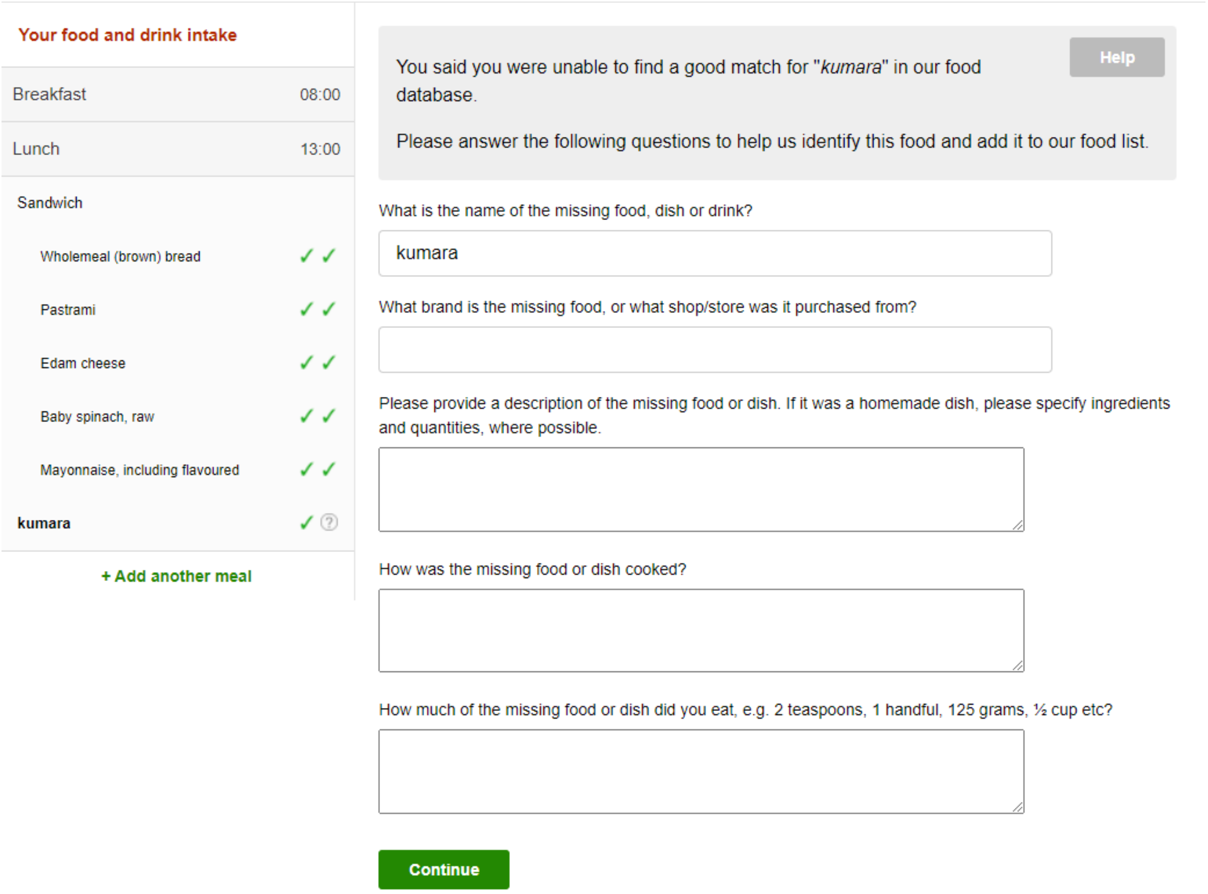

Figure S16. Entering a missing food.

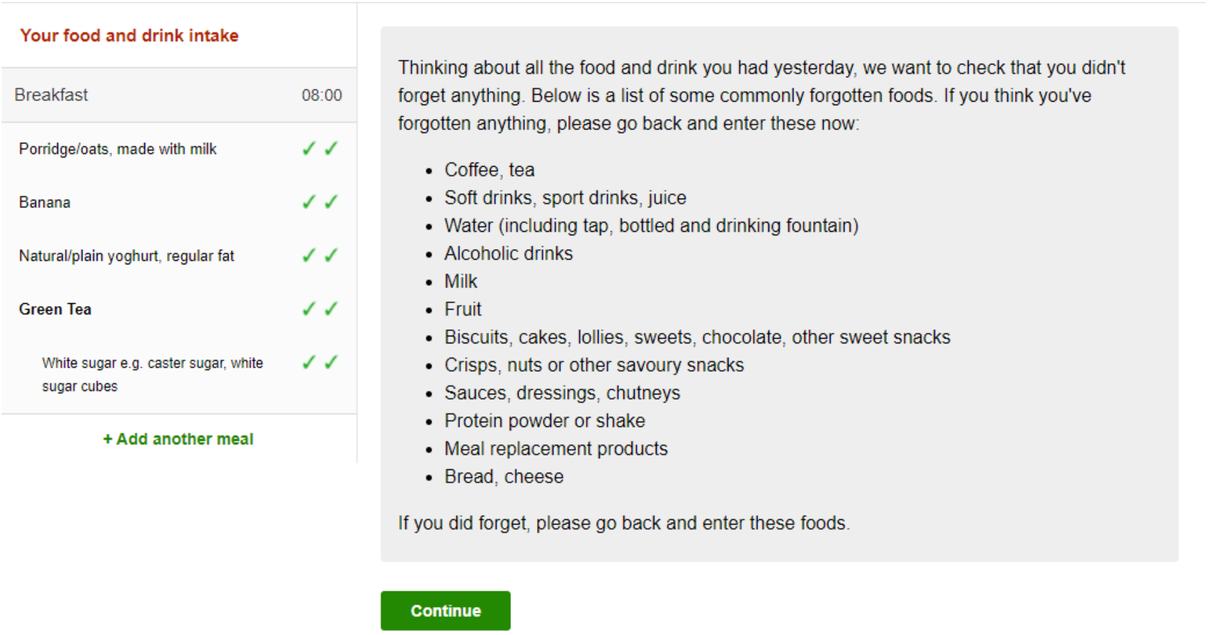

Figure S17. Respondents are prompted to review all food and drinks recorded and add any missing items before submitting the recall.
