# Supplementary material for: Evaluating User Experiences and Preferred Features of a Web-Based 24-Hour Dietary Assessment Tool: Usability Study
Source: JMIR Form Res. 2024 Oct 18;8:e63823. doi: 10.2196/63823 (PMC11530719; doi:10.2196/63823)
Supplement: Multimedia Appendix 2 [file formative_v8i1e63823_app2.pdf]

	Observations	Further details and examples	Implications or consequences
**1**	**Meal menu**		
a	Participants experienced difficulties aligning the default meal menu (ie, breakfast, morning snack/drink, lunch, afternoon snack/drink, evening meal, and late snack/drink) with their meals.	A parent found it difficult to report their child's intake using the default menu, explaining that children do not follow this order (eg, children eat multiple smaller lunches, or consume one meal across multiple eating occasions).<br><br>A participant entered their food in the order that they consumed it and reported their "coffee with milk" as lunch while it was their second morning snack/drink.<br><br>A participant had to delete almost all the meals and snacks they reported as the default meal types and occasions did not match theirs. Changing these took considerable time and the participant commented that it would have been better if they, themselves, could have indicated whether the foods and drinks were part of their breakfast, snack, etc., instead of having a default meal menu.	The observations showed that these issues caused frustration with the participants and misreporting of dietary intake; reporting of duplicate food and drink items, and incorrect timing and meal type.
b	Participants were unsure how to change, remove, or add foods and drinks from the meal menu.	A participant entered seeds and nuts as separate foods (ie, each on a single line). When matching their seeds to a food item from the list, they selected "mixed nuts & seeds". The nuts entry, therefore, became redundant, but the participant was unable to remove this item and reported their mixed nuts twice.	
c	The wording of the "I have finished, continue" button after entering a meal or snack page was confusing.	A participant entered all food and drink items for their breakfast but was unsure what to do next as they did not want to click on the "I have finished, continue" button. Instead, they entered their next meals and snacks by clicking through the left-hand side meal menu (ie, avoiding having to click	Confusion on how to navigate the tool may lead to frustration and longer recall completion times.

	Observations	Further details and examples	Implications or consequences
		on the “I have finished, continue” button). After they reported their last meal, they clicked the “I have finished, continue” button. This took them back through each meal, requiring the participant to click the “I have finished, continue” button several times before continuing to the portion size step.	
**2**	**Food categories**		
a	When unable to find foods or drinks in the initial search results, participants were able to locate the food categories at the bottom of the page and used these to search for their food or drink.	A long list of search results was displayed when a participant searched for “kumara” instead of “kūmara" (macron on ū missing). The participant used the food category “boiled potatoes & kūmara” to find their food.	This is an important feature of the tool that helps participants navigate the food list and, consequently, find their foods and drinks.
b	Food and drink items within the categories were listed in alphabetical order, which participants found easier to navigate and find foods.	Foods and drinks that are similar, but only differ slightly in their description were listed close to each other, which makes recognising the differences easier. A long list of coffees was alphabetically ordered to distinguish between coffees with or without milk, the type of milk, the number of espresso shots, with or without caffeine, and the brewing process.	This could increase the user-friendliness of the tool and is, therefore, an important feature to keep.
c	The list of food and drink items within the food categories was too long to display all items on the participants’ screens.	A participant searched for cabbage using the food category “cabbage & lettuce”. This category included 35 items and was listed in alphabetical order, which resulted in “white/green cabbage” not showing on the page.	This issue prevented the participants from finding and selecting the correct food. Participants reported the food or drink as missing while the item was available in the food list (see observation 4).
**3**	**Salad and sandwich builder**		
a	The option to use the salad or sandwich builder was not always displayed when it would have been appropriate.	Out of nine participants reporting a salad or sandwich, six were not given the option to report this using the builder.<br><br>In many other instances, observations showed that the sandwich builder was not offered when participants entered their sandwich ingredients	These inconsistencies explain the limited use of the builders. The builders prompt participants about commonly used salad or sandwich ingredients to help them remember all items. As such, it is assumed that not using the builders can potentially have a negative impact on the accuracy of the data.

	Observations	Further details and examples	Implications or consequences
		individually (eg, bread, cheese, and tomato as separate items). The software was programmed to recognize the search terms "sandwich" and "salad", and a list of synonyms for these words, which did not include "bread" and "toast".	
b	The salad builder option came up when searching for unrelated foods.	Incorrect showing of the builder option occurred most frequently for the salad builder. Examples of instances when the builder option was displayed included salt, fried rice, salted cashew nuts, cow's milk, rice cake, bliss ball, and pork dumplings.	Although the incorrect showing of the builder may not directly be related to the quality of the dietary recall data, it could possibly confuse participants.
c	Several participants did not notice the salad or sandwich builder button.	Nine participants reported not noticing the builder when they searched for a salad or sandwich (survey findings). At least three of them overlooked this option as the button did appear.	The builder function may not have been clearly visible to the participants and, therefore, overlooked. The builder assists participants in reporting all ingredients in their salad or sandwich, thereby potentially improving the accuracy of the data.
d	The majority of participants who used the salad or sandwich builder were able to navigate through the steps but experienced difficulties finding some foods or had technical issues.	A participant navigated back and forth between the steps of the sandwich builder as they struggled to find the correct foods.<br><br>See observation 6e for an example of a technical issue experienced using the sandwich builder.	Participants did not report all the ingredients as part of their salad or sandwich, which has implications for the accuracy of the dietary data.<br><br>See observation 6e for implications related to technical issues.
**4**	**Missing foods function**		
a	Not all fields were completed in the missing food function.	A participant entered some of the ingredients used for their homemade cheese scroll but left out details about the quantities.	Due to incomplete details, it is impossible to manually record both the type of food and portion size consumed, resulting in missing dietary intake data.
**5**	**Support**		
a	There was some use of the help buttons, and most of the time, this helped the participant resolve their issue.	A participant was confused about the 'same as before' option and clicked the help button to find out more. After reading the information, the participant understood what the question was asking.	The help buttons are an important feature of Intake24-NZ that help participants solve issues, likely having a positive impact on the user experience and accuracy of the data.

	Observations	Further details and examples	Implications or consequences
b	Participants, mostly children and older adults, required support during the recall.	Assistance was provided to navigate Intake24-NZ, find the correct foods and drinks, and solve issues unrelated to the user experience in Intake24-NZ (eg, spelling, recalling details of dietary intake, and sources of meals).<br><br>A parent helped the participant correctly enter their foods in the search bar by telling them to only enter one item on each line and not to include the amount of food.<br><br>There were two instances in which the research team assisted with technical issues.	Individuals may require support to accurately record the foods and drinks consumed and to streamline the dietary recall process.
**6**	**Other**		
a	The instruction video was completely skipped or only partly watched.	Approximately half of the participants watched the full instruction video, while four participants watched part of the video, and nine did not watch it at all.<br><br>Two participants watched the video on 1.25x speed.	As many participants did not watch the instruction video at all or only partly, this may have affected how they used the tool and searched for their foods and drinks, which could subsequently lead to additional issues.
b	Participants were sometimes unsure how to answer the ‘source question’.	Only one out of a list of several sources (eg, supermarket, canteen, food bank, etc.) could be selected to answer the ‘source question’ (ie, “Where was most of the food for this meal purchased or obtained from?”). The participant was unable to select an option from the ‘source question’ when equal amounts of their meal were sourced from different places. The participant entered a protein shake (eg, including protein powder and milk purchased from an online store and supermarket, respectively). Since there were only two ingredients, they found it difficult to	Participants may be unable to accurately answer this question.<br><br>Participants often reported the source of tap water as ‘other’ or selected a random answer to bypass the question.

	Observations	Further details and examples	Implications or consequences
		indicate where the 'majority' of the foods for that meal came from.<br><br>A participant was unsure about the source of their water (from the tap) and asked their parent whether it should be counted as 'homegrown'.	
c	Intake24-NZ functionalities were limited when using a tablet or phone.	The slider function for liquids would not appear when using a tablet.<br><br>Visuals were hard to see, and food items were difficult to change on a phone.	Participants could get confused or frustrated, and they may misreport their intake due to the low user-friendliness when using Intake24-NZ on a tablet or phone.
d	Some participants struggled with the (24-hour) time indication.	A participant tried to enter the time of their breakfast by typing it instead of using the up and down arrow buttons and ultimately was not able to report the exact time.<br><br>A participant was unsure how to read the 24-hour time and needed support converting 2 PM to 14:00.	At times, this may lead to (slight) misreporting of the timing of the meals consumed.
e	Several technical issues occurred.	Intake24-NZ froze and did not let the participant continue to the next step.<br><br>When entering a sandwich in the builder, the participant unintentionally selected lettuce and mayonnaise multiple times after the system kept looping them back to the same page.	Depending on the nature of the technical issues, these can lead to multiple unwanted implications, such as partially completed recalls or inaccurate reporting of dietary data.

NZ: New Zealand
